# Changes in EBV Association Pattern in Pediatric Classic Hodgkin Lymphoma From a Single Institution in Argentina

**DOI:** 10.3389/fonc.2019.00881

**Published:** 2019-09-18

**Authors:** Elena De Matteo, Mercedes García Lombardi, Maria V. Preciado, Paola Chabay

**Affiliations:** ^1^Multidisciplinary Institute for Investigation in Pediatric Pathologies (IMIPP), CONICET-GCBA, Pathology Division, Ricardo Gutiérrez Children's Hospital, Buenos Aires, Argentina; ^2^Oncology Division, Ricardo Gutiérrez Children's Hospital, Buenos Aires, Argentina; ^3^Multidisciplinary Institute for Investigation in Pediatric Pathologies (IMIPP), CONICET-GCBA, Molecular Biology Laboratory, Pathology Division, Ricardo Gutiérrez Children's Hospital, Buenos Aires, Argentina

**Keywords:** Epstein Barr Virus, Hodgkin lymphoma, children, epidemiology, nodular sclerosis

## Abstract

In classic Hodgkin lymphoma (cHL), Epstein Barr virus (EBV) association varies worldwide.

**Aims:** Our aim was to analyze EBV association with pediatric cHL for the last 28 years.

**Methods:** EBV presence was evaluated by EBERs *in situ* hybridization and LMP1 immunohistochemistry.

**Results:** Until 2008, we found in pediatric cHL a similar percentage of EBV presence to those observed in adult cHL from developed populations. Nevertheless, in the last 8 years, an unexpected difference in cHL EBV association was proven, along with a slight bias of EBV association with the nodular sclerosis (NS) subtype. Concerning histological subtype distribution, even though MC still prevailed in the whole series, those cases diagnosed as NS showed a sustained rise from 1989 until today.

**Conclusion:** Variations of EBV association of cHL related to geography, age, ethnicity, and histological type have been largely described when compared with different world regions, but interestingly, this single-center revised series brought to light the dynamic process behind the evolution of this relationship over time.

## Introduction

Epstein Barr virus (EBV) is related to more than 200,000 cases of cancer every year; moreover, 1.8% of all cancer deaths are caused by EBV-related neoplasias^1^. The three major types of B cell malignancies associated to EBV are Burkitt, classic Hodgkin, and diffuse large B cell lymphomas (BL, cHL, and DLBCL), in which EBV presence varies from 10 to 100% of cases in BL, ~50% in cHL, and ~10% in DLBCL. In addition, EBV is associated in 100% of cases with T/NK lymphoproliferations and nasopharyngeal carcinoma, as well as in ~10% in gastric carcinoma (1). Nevertheless, EBV association is not a requisite for them to occur as denoted by their occurrence, in variable proportion, in the absence of EBV as well (2). CHL is subclassified into nodular sclerosis (NS), mixed cellularity (MC), lymphocyte-rich (LRHL), and lymphocyte-depleted (LDHL) (3). CHL includes three different disease entities: pediatric HL (EBV-positive, mixed cellularity subtype), HL in young adults (EBV-negative, nodular sclerosis subtype), and HL in older adults (EBV-positive, mixed cellularity subtype) (4). In European and North American reports, tumor cells are infected by EBV in ~40% of cHL cases (5). However, EBV association with cHL shows a different age distribution in underdeveloped or developing populations, mostly as a consequence of socioeconomic status, but also attributable to other factors, such as race or ethnicity, suggesting an interplay between environmental and genetic factors related to the etiology of EBV-positive cHL. In fact, it was demonstrated that EBV presence in US- and foreign-born Hispanics was increased in patients aged 0–14 years and older than 55 years, while among whites, EBV-positive cases were observed mostly in patients older than 40 years (6). However, in a series of pediatric cHL from Brazil, EBV-associated cases were not specifically found in younger patients (7).

Racial/ethnic differences showed higher HL rates and lower EBV+ tumor prevalence in whites. In fact, Glaser et al. compared EBV-associated HL rates in US Hispanics with non-Hispanic whites, and reported and EBV prevalence of 67, 32, and 23% among foreign-born Hispanics, US-born Hispanics, and whites, respectively, revealing how environment affects HL incidence variation (6). Childhood cHL represents 6% of all cancers in the 0–14 years age group with a typical male preponderance. The MC histological subtype is the one mostly associated with EBV, prevails in young children, and represents around 20% of HL, while the NS subtype is mostly seen in adolescents and young adults and denotes about 75% of HL. Tumor cells are EBV positive in ~30 and 90% of all pediatric HL cases in developed and underdeveloped or developing countries, respectively (8).

Those facts clearly demonstrated that EBV association rates of cHL vary among populations, based on differences in both environmental and genetic features. Socioeconomical factors, such as poverty, child mortality, illiteracy, unsatisfied basic needs, and running water, may fluctuate in countries like Argentina during a long time period, showing evidence of a dynamic scenario. Our previous work, which included pediatric pre-operative serum samples from patients younger than 15 years old, demonstrated that 72% of children were seropositive for EBV by the age of 3 years (9). Therefore, since no previous studies have examine temporal trends of cHL in an Argentine pediatric cohort, our aim was to analyze the course of pediatric cHL features in a single institution for the last 28 years, particularly in relation to EBV.

## Materials and Methods

### Patients and Samples

Formalin-fixed paraffin-embedded (FFPE) biopsy samples from 174 patients were collected from the archives at Pathology Division, Ricardo Gutierrez Children's Hospital, a national referent institution that receives cases from the whole country, from 1989 to 2017. Diagnosis was performed from primary biopsies according to the WHO scheme (10). Institutional guidelines regarding human experimentation in accordance with the Helsinki Declaration of 1975 were followed. The Ethical Committee of the Ricardo Gutierrez Children Hospital has approved the study, and patients' guardians provided written informed consent in accordance with the Declaration of Helsinki.

### EBV Analysis

EBERs *in situ* hybridization (ISH) was performed on the 174 FFPE tissue sections using fluorescein isothiocyanate (FITC)-conjugated EBERs oligonucleotides as probes (Dako) according to the manufacturer's instructions. A monoclonal antibody anti-FITC labeled with alkaline phosphatase was used for the detection of hybridized sites (Dako). An EBV-associated post-transplant lymphoproliferative disorder was used as a positive control.

Immunostaining using monoclonal antibodies CS1-4 (Dako) was performed to localize LMP1 expression. Primary antibodies were incubated overnight at 4°C. IHC detection of primary antibodies was carried out using a universal streptavidin–biotin complex-peroxidase detection system UltraTek HRP Anti-Polyvalent Lab Pack (ScyTek) as previously described (11). An EBV-associated post-transplant lymphoproliferative disorder biopsy was used as a positive control, and a negative control was performed without the primary antibody.

### Statistical Analysis

Categorical variables (EBV presence, age group, gender, HL subtypes) were analyzed using Fisher's exact test. Mann–Whitney test was used to compare the age means in relation to EBV presence and histological subtypes. The Cochran–Armitage test was used to assess differences in the percentage of EBV positive cases, or subtype distribution during the five periods. Survival distributions were estimated with the Kaplan–Meier method, and the differences were compared by the log-rank test. All tests were two-tailed, and a *p* < 0.05 was considered statistically significant.

## Results

EBV association with cHL was observed in 103/174 patients (59%) by EBERs ISH and LMP1 expression. As expected, EBV was statistically associated to MC subtype since it was observed in 65/84 (77%) of cases, compared to 25/65 (38%) in NS, 10/20 (50%) in LRHL, and 3/5 (60%) in LDHL (*p* < 0.0001, Fisher exact test). In addition, EBV presence was statistically associated with patients younger than 10 years old (68% of EBV+ cases) (*p* = 0.0003, Fisher exact test) and males (98% of EBV+ cases) (*p* = 0.009, Fisher exact test).

When EBV association was analyzed by discriminating five periods, unexpectedly, in the last 8 years, a slight increase in EBV association was observed, given that the 2009–2013 and 2014–2017 periods displayed an EBV positivity of 65 and 72%, respectively (Table 1), although this increase was not statistically significant (*p* = 0.169, Cochran–Armitage test for trend). When analyzing cHL distribution according to age, an increase in the median age on EBV-associated cases over time was observed (Supplementary Table 1), but without statistical significance when EBV+ cases were compared (*p* = 0.1555, Kruskal–Wallis test). Finally, when histological subtypes were analyzed in relation to age in the 28-years period, MC diagnosis turned out to be statistically associated with younger patients (median age MC 7 years, NS 11 years, LRHL 11 years, and LDHL 10 years, *p* = 0.0006, Kruskal–Wallis test).

**Table 1:** EBV positivity in pediatric cHL according to MC and NS subtypes.

	**All subtypes**			**MC**		**NS**			
**Year range**	**EBV+/total**	**%**	***p***	**EBV+/total**	**%**	***p***	**EBV+/total**	**%**	***p***
1989–1993	16/29	55		13/18	72		0/5	0	
1994–1998	17/34	50		12/18	67		3/11	27	
1999–2003	28/44	64		19/21	90		7/20	35	
2004–2008	7/15	47		2/6	33		3/6	50	
2009–2013	22/34	65		12/14	86		9/15	60	
2014–2017	13/18	72		7/7	100		3/8	38	
*Total*	*103/174*	*59*	*0.169*	*65/84*	*77*	*0.180*	*25/65*	*38*	0.041^*^

MC, mixed cellularity; NS, nodular sclerosis. p-value as determined by Cochran–Armitage test for trend.

* *p < 0.05*.

In order to explain the slight increase in EBV positivity, viral association was analyzed deeply throughout the five periods specifically in both MC and NS prevalent subtypes. Interestingly, EBV association with NS subtype showed a statistical difference over time, showing a peak of 60% in the 2009–2013 period (*p* = 0.041, Cochran–Armitage test for trend), while MC subtype showed similar percentages of association with EBV throughout the analysis (*p* = 0.180, Cochran–Armitage test for trend) (Table 1). At this point, the question that arises is if this difference could be a consequence of an increase in the incidence of NS subtype itself. Even though the MC subtype still prevails in the whole series, the proportion of cases diagnosed as NS subtype showed a statistical increase from 1989 (17%) until today (42%) (*p* = 0.024, Cochran–Armitage test for trend), compared with the MC subtype (Supplementary Table 2).

**Figure 1:**
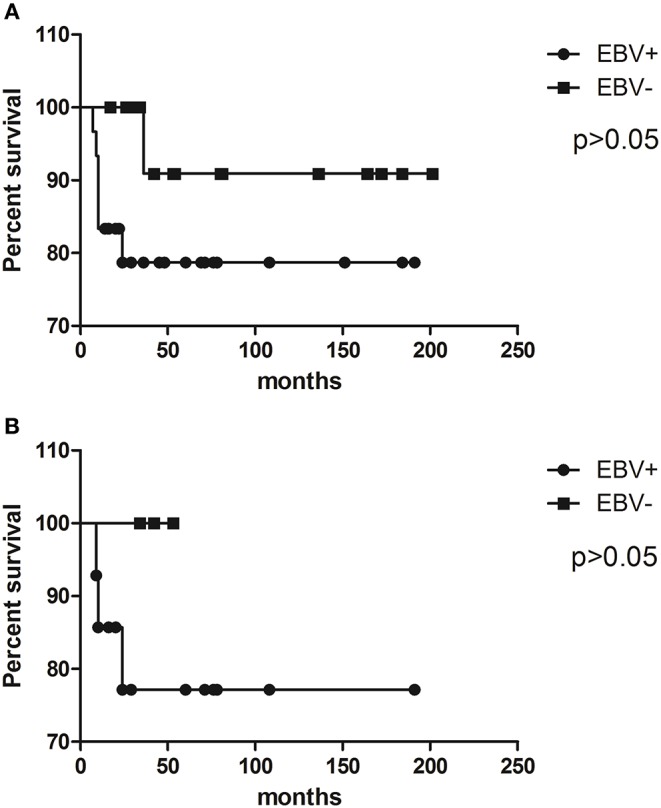
Kaplan–Meier survival curves illustrate event-free survival (EFS) analysis of pediatric cHL cohort related to EBV status in **(A)** the whole cohort and **(B)** patients younger than 10 years old.

In our whole pediatric cHL series, EBV status had no significant influence on prognosis (*p* = 0.2093, log rank test) (Figure 1A). Given the fact that EBV has a higher incidence of cHL among patients under 10 years old, survival analysis was restricted to this specific group, but the virus did not determine survival as well (*p* = 0.3883, log rank test) (Figure 1B).

## Discussion

In our cohort, EBV positivity displayed similar percentages to those observed in developed populations (~50%), but lower than the previously described for underdeveloped countries (~90%) (3). Regardless of an HL–EBV association resembling developed countries, the virus was statistically associated with HL cases occurring in children younger than 10 years, such as in underdeveloped countries.

Concerning histological subtypes, childhood cHL typically shows a significant prevalence of MC. In order to further characterize EBV distribution in HL subtypes, we discriminate viral presence in both major subtypes, MC and NS. As a matter of fact, EBV association with the NS subtype showed a difference in the last years. We suggest that the shift to NS diagnosis in our pediatric cHL may contribute to the detection of more EBV-related NS cases and also might explain the higher EBV presence in our population in the last years. Several socioeconomic status indicators in Argentina improved during the last two decades, such as poverty, child mortality, illiteracy, unsatisfied basic needs, and running water (12), which possibly could explain the lower cHL incidence along with the shift toward NS subtype, resembling developed populations. Nevertheless, EBV infection status was not influenced by this improvement in our series, given that EBV association with pediatric cHL did not exhibit any changes in children from our institution. On the other hand, in children from Brazil, EBV-associated cHL decreased from 96 to 64% over the last 54-years period (13), whereas the same finding was demonstrated in children from Korea, in which a decrease from 21 to 8.6% was proven (14). An improvement in socioeconomic conditions in these two populations was suggested to be related to the change toward a pattern of EBV association similar to developed populations (13).

The identification of prognostic biomarkers in children that correlates with clinical outcome is important, especially for those patients with relapse or refractory disease (8). The prognostic significance of EBV in pediatric patients with cHL is still controversial. In a large study performed by Claviez et al. in pediatric cHL with combined treatment modalities, in a univariate analysis, EBV infection has no influence on overall survival (OS), but it is associated with an inferior OS in specific subgroups, such as patients with NS and increased numbers of Hodgkin Reed Sternberg (HRS) cells. However, LMP1 presence in HRS cells was proved to be an independent prognostic factor associated with an inferior OS, with no effect on failure free survival (FFS) (15). In contrast, several groups reported no association between EBV and clinical outcome (16), or better prognosis in EBV+ cases (17). Different outcomes might also be a consequence of variations in the presence of EBV+ HL that is related to geography, age, ethnicity, and histological type (14). Therefore, analyses of EBV association with HL in specific populations are mandatory. In our whole pediatric cHL population, even if EBV seems to have an important role in cHL pathogenesis in young children, it has no impact on survival.

In summary, in the last 30 years, we have observed an increase in NS subtype in pediatric cHL from our institution, along with a difference in the incidence of EBV in this subtype. Even the fact that EBV association with pediatric cHL in Argentina is still similar to that observed in developed populations, the shift of EBV presence in young children with NS cHL may suggest a detrimental influence of early-life exposure to viral infection. Improvement of socioeconomic conditions did not show a positive impact in EBV-associated cHL in our series, suggesting that others factors might be involved in viral pathogenesis. These findings reveal the need for more studies involving pediatric groups from underdeveloped regions to disclose the complexity of EBV involvement in the lymphomagenesis process in pediatric patients.

## Data Availability

All datasets generated for this study are included in the manuscript.

## Ethics Statement

The studies involving human participants were reviewed and approved by The Ricardo Gutierrez Ethical Committee and patients' guardians gave written informed consent in accordance with the Declaration of Helsinki. Written informed consent to participate in this study was provided by the participants' legal guardian/next of kin.

## Author Contributions

ED acquired and analyzed the data. MG analyzed clinical data and survival. MP critically revised the manuscript for important intellectual content. PC designed and analyzed research, performed statistical analysis, and wrote the manuscript.

### Conflict of Interest Statement

The authors declare that the research was conducted in the absence of any commercial or financial relationships that could be construed as a potential conflict of interest.
