# Supplementary material for: Changes in EBV Association Pattern in Pediatric Classic Hodgkin Lymphoma From a Single Institution in Argentina
Source: Front Oncol. 2019 Sep 18;9:881. doi: 10.3389/fonc.2019.00881 (PMC6759519; doi:10.3389/fonc.2019.00881)
Supplement: Supplementary file 1 [file Table_1.DOCX]

Supplementary Table 1: Age distribution in the whole period according to EBV presence

| ***Year range*** | Median age  total cases | Median Age  EBV+ cases | Median age  EBV- cases | p |
| --- | --- | --- | --- | --- |
| 1989-1993 | 8 | 6 | 11 | 0.0314^*^ |
| 1994-1998 | 8 | 7 | 12 | 0.0590^#^ |
| 1999-2003 | 9 | 8 | 12 | 0.0772^#^ |
| 2004-2008 | 10 | 10 | 10 | 0.6428 |
| 2009-2013 | 11 | 8 | 13 | 0.0322^*^ |
| 2014-2017 | 12 | 14 | 11 | 0.9651 |

p value as determined by Mann Whitney test. * p<0.05, ^#^ p value indicates trend.

Supplementary Table 2: cHL subtypes distribution in the whole period

| ***Year range*** | **MC/ total cases (%)** | **NS/ total cases (%)** | **LRHL/ total cases (%)** | **LDHL/ total cases (%)** |
| --- | --- | --- | --- | --- |
| 1989-1993 | 18/29 (62) | 5/29 (17) | 5/29 (17) | 1/29 (3) |
| 1994-1998 | 18/33 (55) | 11/33 (33) | 3/33 (9) | 1/33 (3) |
| 1999-2003 | 21/44 (48) | 20/44 (45) | 1/44 (2) | 2/44 (5) |
| 2004-2008 | 6/15 (40) | 6/15 (40) | 3/15 (20) | 0/15 (0) |
| 2009-2013 | 14/34 (41) | 15/34 (44) | 4/34 (12) | 1/34 (3) |
| 2014-2017 | 7/19 (37) | 8/19 (42) | 4/19 (21) | 0/19 (0) |
| ***Total*** | **84/174 (48)** | **65/174 (37)** | **20/174 (11)** | **5 /174 (3)** |

Abreviations: MC, mixed cellularity; NS, nodular sclerosis; LRHL, lymphocyte rich Hodgkin lymphoma; LDHL, lymphocyte depletion Hodgkin lymphoma.
